# Editorial: Load and wellness monitoring in sports: the relationship between different metrics

**DOI:** 10.3389/fspor.2025.1570314

**Published:** 2025-03-03

**Authors:** R. Oliveira, J. P. Brito, F. T. González-Fernández, R. Morgans

**Affiliations:** ^1^ Research Centre in Sports Sciences, Health and Human Development, (CIDESD), Santarém Polytechnic University, Rio Maior, Portugal; ^2^ Santarém Polytechnic University, School of Sport, Rio Maior, Portugal; ^3^ Department of Physical Education and Sports, University of Granada, Granada, Spain; ^4^ School of Sport and Health Sciences, Cardiff Metropolitan University, Cardiff, United Kingdom

**Keywords:** fitness, injury, load, sports, performance, wellbeing

**Editorial on the Research Topic**
Load and wellness monitoring in sports: the relationship between different metrics

## Introduction

Load and wellness (sometimes referred to as well-being) monitoring of training or competition is useful for improving performance (1). While load can be divided into two dimensions: internal [e.g., ratings of perceived exertion (RPE) and heart rate] and external (e.g., running and accelerometer-based measures) (2, 3), wellness can also include various measures of fatigue, sleep quality, muscle soreness, stress, and mood which are regularly collected through questionnaires (4, 5).

However, the existing literature is not consistent in demonstrating the relationship between load and wellness metrics. For example, a systematic review investigated the relationship between wellness and training load measures in team sport athletes, revealing a spectrum of relationships ranging from no association to very strong associations (6). Moreover, a more recent systematic review suggested that poor sleep quality and quantity might negatively affect soccer performance while increasing the risk of injury (7). Furthermore, Rico-González et al. (8) examined team sports (rugby, handball, basketball, futsal, hockey, and Australian football) and observed that Immunoglobulin A (IgA) tended to decrease with increased training load or high demand periods. An association was found between low levels of IgA and higher upper respiratory tract infections, which may result in decreased wellness (8).

Therefore, the aim of this research topic was to provide information on load and wellness monitoring of different sports and analyze the relationship between the different dimensions.

### Contributing articles

The articles in this Research Topic covered diverse aspects of sports such as: the prediction of soccer injuries using GPS-based metrics (e.g., acceleration and deceleration); the impact of sleep on emotional and physical well-being, using daily sleep diaries and questionnaires in professional cricketers; the stretch-shortening cycle fatigue response, assessed through countermovement jumps and 10-5 hop tests, to a high-intensity stress phase of training in collegiate men's basketball; the internal load, measured through energy expenditure, oxygen consumption, heart rate and RPE, of seven different indoor cardiovascular machines; the training intensity distribution of the different training zones (i.e., zone 1, 2 and 2), evaluated through different internal load measures [heat rate, blood lactate, peak oxygen consumption (VO_2peak_) and RPE] and external load measures (% of running pace, power, velocity) of elite-to-world-class endurance athletes; and the analysis of sports initiation (M.A.M.I.deporte®) practiced by between children and parents through a survey analysis.

In this Research topic there are three articles on sports, two articles examining recreational participants and one review. Among the articles on sports, Saberisani et al. found that no external load variables showed high predictive power (>0.95), although a close value (0.91) was observed for the number of decelerations, highlighting the load management of this metric as a potential injury risk factor in soccer. The same authors also suggested using this metric for acute, chronic, and acute-to-chronic workload calculations, which can be replicated for different soccer contexts (e.g., different countries, leagues, ages and sexes) and other team sports (e.g., futsal, handball, basketball, rugby, etc.). Grewal et al. focused their study on wellness, specifically sleep (although also included were muscle soreness, readiness, stress and fatigue monitoring). Their research highlighted that better sleep quality positively influences the overall emotional and physical well-being of professional cricketers. Their results emphasize the importance of targeted sleep interventions to improve sleep quality and subsequently optimize psychological and physiological wellness. Furthermore, the findings of the article can be replicated in different contexts of both individual and team sports. Meanwhile, Philipp et al. explored the stretch-shortening-cycle fatigue response (through different types of jumps such as countermovement jumps and 10-5 hops) to a one-week-long high-intensity fatiguing phase of training and found that basketball players experienced acute fatigue along with potentially longer-lasting reductions in performance. Notably, these findings can be replicated and also highlight the practical ease of these tests to monitor fatigue in various contexts.

Focusing on recreational participants, Prieto-González et al. compared energy expenditure, VO_2peak_, and heart rate recorded in middle-aged adults exercising on seven different indoor cardiovascular machines at maximal and submaximal intensities. The main findings showed that the treadmill provided the greatest energy expenditure, followed by the stair climber and the elliptical trainer. It was also reported that participants displayed greater enjoyment which may lead to improved exercise adherence. Additionally, a better understanding of the influence of parents on children's physical activity is crucial to improve and increase the positive effects on health and well-being.

Finally, Sperlich et al. presented a review of the training intensity distribution [training at moderate (Zone 1), heavy (Z2) and severe (Z3)] of elite-to-world-class endurance athletes during different phases of the season and highlighted that the existing literature does not allow general conclusions to be drawn due to the wide variability of the utilized methods. Furthermore, the lack of contextual information regarding the mode of exercise, environmental conditions, and biomechanical aspects of the exercise is evident. Such findings reinforce the inclusion of both the type of load quantification (external and internal) and wellness monitoring for better insights and load adjustments. Furthermore, the study context, participants and exercise protocols are essential for improving research methods and their replicability.

## Conclusion

The articles presented in this Research Topic contribute to a better understanding of the interaction of various load and wellness metrics in different sports. With the exception of the review, the other studies presented here can be fully replicated in other sports settings or geographic contexts, potentially validating the findings discussed. This compilation addresses multiple sports and includes young, professional, recreational, male, and female athletes. Importantly, it addresses some gaps in the literature such as the simultaneous practice of physical activity by parents and their children. Consequently, this Research Topic serves as a valuable tool for sports researchers, coaches, parents, and practitioners.

Nonetheless, more research is needed to provide deeper insights into training methodologies and to clarify when to use different metrics and how to interpret them.
